# Genetics and Clinical Characteristics of PPARγ Variant-Induced Diabetes in a Chinese Han Population

**DOI:** 10.3389/fendo.2021.677130

**Published:** 2021-10-26

**Authors:** Siqian Gong, Xueyao Han, Meng Li, Xiaoling Cai, Wei Liu, Yingying Luo, Si-min Zhang, Lingli Zhou, Yumin Ma, Xiuting Huang, Yufeng Li, Xianghai Zhou, Yu Zhu, Qiuping Wang, Ling Chen, Qian Ren, Ping Zhang, Linong Ji

**Affiliations:** ^1^ Department of Endocrinology and Metabolism, Peking University People’s Hospital, Peking University Diabetes Center, Beijing, China; ^2^ Department of Endocrinology, Beijing Pinggu District Hospital, Beijing, China; ^3^ Department of Endocrinology, Beijing Liangxiang Hospital, Beijing, China

**Keywords:** type 2 diabetes, activation function 1 domain, lipodystrophy, Tyr95Cys, PPARG, prevalence

## Abstract

**Objectives:**

*PPARγ* variants cause lipodystrophy, insulin resistance, and diabetes. This study aimed to determine the relationship between *PPARγ* genotypes and phenotypes and to explore the pathogenesis of diabetes beyond this relationship.

**Methods:**

*PPARγ2* exons in 1,002 Chinese patients with early-onset type 2 diabetes (diagnosed before 40 years of age) were sequenced. The functions of variants were evaluated by *in vitro* assays. Additionally, a review of the literature was performed to obtain all reported cases with rare *PPARγ2* variants to evaluate the characteristics of variants in different functional domains.

**Results:**

Six (0.6%) patients had *PPARγ2* variant-induced diabetes (PPARG-DM) in the early-onset type 2 diabetes group, including three with the p.Tyr95Cys variant in activation function 1 domain (AF1), of which five patients (83%) had diabetic kidney disease (DKD). Functional experiments showed that p.Tyr95Cys suppresses 3T3-L1 preadipocyte differentiation. A total of 64 cases with damaging rare variants were reported previously. Patients with rare *PPARγ2* variants in AF1 of *PPARγ2* had a lower risk of lipodystrophy and a higher rate of obesity than those with variants in other domains, as confirmed in patients identified in this study.

**Conclusion:**

The prevalence of PPARG-DM is similar in Caucasian and Chinese populations, and DKD was often observed in these patients. Patients with variants in the AF1 of *PPARγ2* had milder clinical phenotypes and lack typical lipodystrophy features than those with variants in other domains. Our findings emphasize the importance of screening such patients *via* genetic testing and suggest that thiazolidinediones might be a good choice for these patients.

## Introduction

Peroxisome proliferator-activated receptor γ (PPARγ) is a ligand-dependent transcription factor with key roles in adipocyte differentiation, adipogenesis, glucose homeostasis, and inflammation (1). Several rare *PPARγ* variants have been reported as causes of familial partial lipodystrophy (FPLD3, OMIM 604367), insulin resistance, diabetes, and hypertriglyceridemia. In some patients with FPLD3, thiazolidinediones effectively ameliorate hyperglycemia and insulin resistance (2). However, cases with rare *PPARγ* variants have not been reported in the Chinese population. Since thiazolidinedione-based precision medicine may be beneficial for patients with *PPARγ* variant-induced diabetes (PPARG-DM), it is necessary to determine the prevalence of PPARG-DM and develop clinical strategies for screening this condition.

There are four functional domains in PPARγ, including the activation function 1 (AF1) domain, DNA-binding domain (DBD), hinge domain (HD), and ligand-binding domain (LBD). Two isoforms of PPARγ (PPARγ1 and PPARγ2) differ with regard to the N-terminal region. An additional 30 amino acids in N-terminal ligand-independent AF1 of *PPARγ*2 results in a 5–10-fold increase in the activation function *in vitro* (3). PPARγ2 plays a more dominant role in glucose and lipid metabolism than PPARγ1 (4). The AF1 of PPARγ is involved in PPAR signaling *via* posttranslational modifications, such as phosphorylation or SUMOylation, and interactions with co-factors (5). A study has shown that a polymorphism in the AF1 domain of *PPARγ2* (NP_056953.2: p.Pro12Ala) is associated with type 2 diabetes (T2DM) (6). Patients carrying a rare variant, such as p.Pro113Gln, in the AF1 of *PPARγ2* show marked obesity and/or hyperglycemia (7). In contrast, patients with rare variants in domains other than AF1 (non-AF1) consistently show a reduced fat mass and severe insulin resistance. Thus, we hypothesize that the effects of *PPARγ2* variants depend on the location of the variants.

Studies have shown that the p.Pro12Ala variant in *PPARγ2* is associated with diabetic kidney disease (DKD) (8, 9), and this was supported by an animal study showing that mice with the *PPARγ* knockout develop DKD (10). However, the association between DKD and p.Pro12Ala has not been validated in Asian populations (11, 12). Furthermore, renal phenotypes for different rare *PPARγ2* variants have not been fully characterized.

In this study, we estimated the prevalence of PPARG-DM in the Chinese population, confirmed the relationship between disease phenotypes and variants in different domains, and explored the pathogenesis of PPARG-DM beyond this relationship. Furthermore, we reviewed all reported cases with rare *PPARγ2* genotypes in the literature and compared the clinical phenotypes of individuals with *PPARγ2* variants in different domains.

## Materials and Methods

### Study Overview

**Figure 1:**
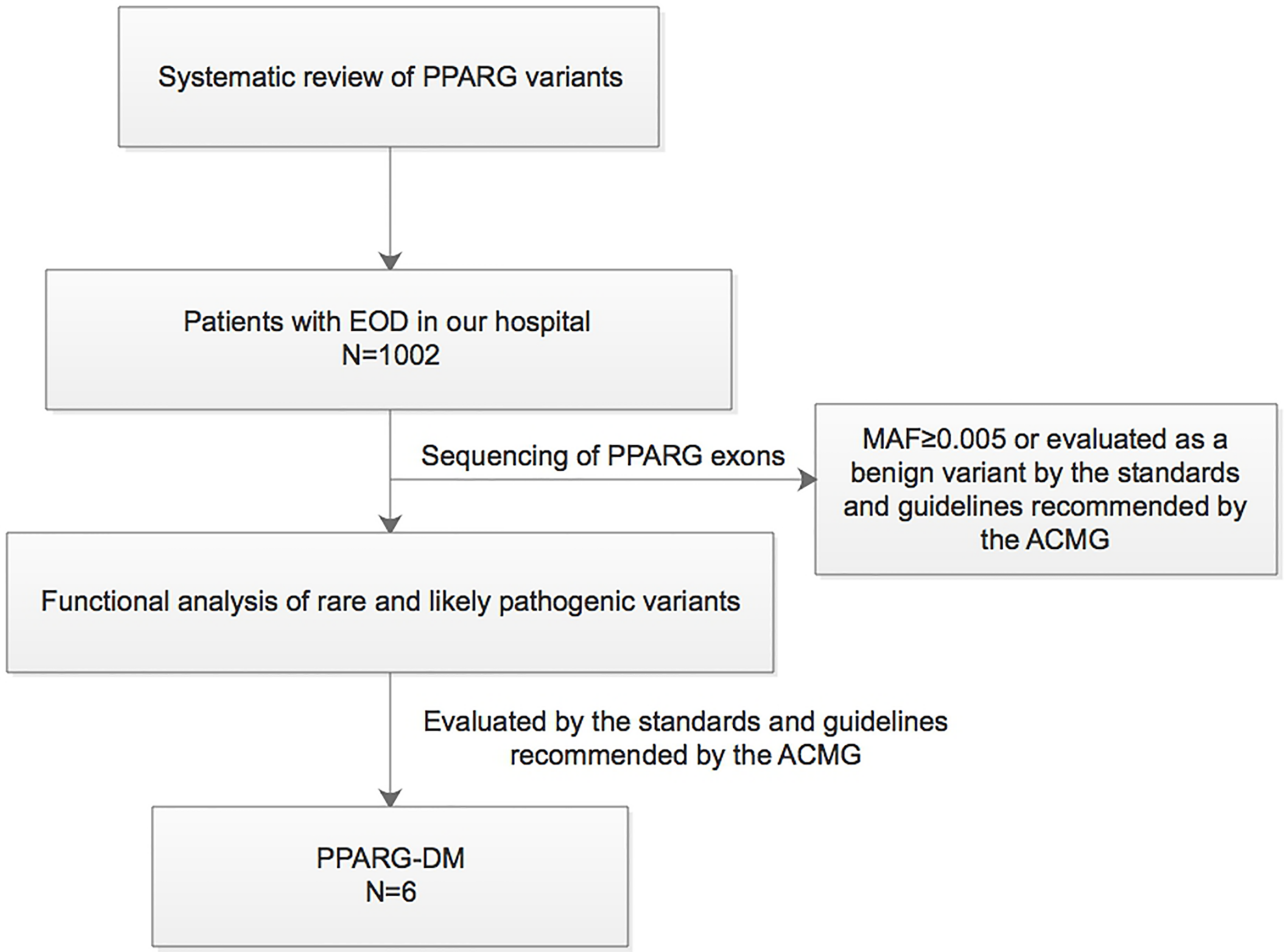
Synopsis of this study. EOD, early-onset type 2 diabetes; MAF, minor allele frequency; PPARG-DM, PPARγ variant-induced diabetes; ACMG, American College of Medical Genetics.

This study was conducted in compliance with the Declaration of Helsinki. The study protocol was approved by the Ethics Committee of Peking University People’s Hospital (2014-06, 2017PHB035-01). Written informed consent was obtained from all the study participants. A synopsis of the study is shown in **Figure 1**.

### *PPARγ2* Gene Sequencing of Patients With Early-Onset Diabetes (Cohort 1)

A total of 1,002 unrelated Chinese patients (age ≥ 18 years) with early-onset diabetes (EOD), who had been diagnosed with T2DM before the age of 40 years in the Endocrinology and Metabolism Department of the Peking University People’s Hospital between June 2014 and October 2018, were included. Diabetes was diagnosed according to the 1999 WHO criteria (13). Patients with typical clinical features of type 1 diabetes or other specific forms of diabetes (e.g., chronic pancreatitis) and those who were positive for anti-glutamic acid decarboxylase antibodies, anti-islet cell antibodies, or anti-insulin antibodies (among individuals not receiving insulin) were excluded from this study.

### Biochemical Measurements and Clinical Information

The clinical characteristics of patients were collected at the time of enrollment. Laboratory tests and comorbidities were defined as described previously (14). The parameters were defined as follows: HbA_1c_ National Glycohemoglobin Standardization Program/International Federation of Clinical Chemistry and laboratory medicine (NGSP/IFCC) conversion calculated the following: NGSP (%) = IFCC (mmol/mol) × 0.0915 + 2.152. Homeostasis model assessment (HOMA) was used to evaluate β-cell function and insulin sensitivity. Insulin resistance index (HOMA-IR) = [Fasting insulin (Fins, mIU/L) × fasting plasma glucose (FPG, mmol/L)]/22.5, and β-cell function (HOMA-β) = [Fins (mIU/L) × 20]/[FPG (mmol/L) − 3.5 (%)] (15). Estimated glomerular filtration rate (eGFR, mL/min/1.73 m^2^) = 175 × Serum creatine (Scr, mg/dl)^−1.234^ × age (years)^−0.179^ × 0.79 (if female) (16). DKD was defined as a urinary albumin/creatinine ratio (UACR) ≥30 mg/g and/or eGFR <60 ml/min/1.73 m^2^. Serum adiponectin (EHC120; NeoBioscience, Shanghai, China) and serum leptin (EZHL-80SK; EMD Millipore, Burlington, MA, USA) levels were determined by ELISA. Serum leptin levels were measured in 85 subjects and the reference range was determined. These subjects had normal glucose tolerance (NGT) and HbA_1c <_6% and normal BMI, lipid levels, and kidney and liver function, as described previously (17). The 90% interval was 0.49–5.41 ng/ml for men and 2.57–15.75 ng/ml for women.

### Sequencing of Exons in *PPARγ2*

All DNA samples were extracted from peripheral blood. Next-generation sequencing (*n* = 541) and Sanger sequencing (*n* = 461) were used for the identification of variants in *PPARγ2* exons. For Sanger sequencing, an Applied Biosystems 2720 Thermal Cycler (Waltham, MA, USA) was used [the primers used are shown in the **Electronic Supplementary Material (ESM) Table 1**]. For next-generation sequencing, the Roche NimbleGen Human Exon V2 capture chip was used, and sequencing was performed on the Illumina HiSeq2500 system. Sequencing data were obtained with a target gene coverage (~100×) of >99% and a depth of >200 bp. The quality scores for all FASTQ data were Q20 >90% and Q30 >85%. All rare variants of *PPARγ2* were validated by Sanger sequencing.

### Functional Analysis of Rare Variants of *PPARγ2* Identified in This Study

The *PPARγ*2 wild-type construct was prepared by subcloning the full-length human *PPARγ*2 cDNA (NM_015869) in frame into the *Xho*I and *Kpn*I sites of the GV141 vector (GeneChem, Shanghai, China) (**ESM Figure 1**). The p.Glu217Lys (E217K), p.Ser186Gly (S186G), p.Tyr95Cys (Y95C), and p.Ile264Thr (I264T) variants in *PPARγ*2 were generated by PCR, digested with *Xho*I and *Kpn*I, and ligated into the GV141 plasmid. Both the wild-type and mutant clones were fully sequenced. All constructs were verified by direct sequencing.

293T cells were cultured in Dulbecco’s modified Eagle medium (DMEM) (11995065; Invitrogen, Waltham, MA, USA) containing 10% fetal bovine serum (Gibco, Invitrogen) at 37°C on 24-well plates. Cells were grown to 60% confluence for transient transfection using X-tremeGENE HP (06366236001; Roche, Basel, Switzerland). Transfection efficiency had been evaluated by a fluorescent microscope. Luciferase activities of E217K, Y95C, S186G, and I264T, relative to those of wild-type PPARγ2, were evaluated using the Dual-Luciferase Reporter Assay System (E1910; Promega, Madison, WI, USA). The media did not contain any PPARγ agonist. The 293T cells were co-transfected with a REPO PPARγ reporter (Genomeditech, Shanghai, China, **ESM Figure 2**) and the constructed vectors to assay the PPARγ activity. Data are presented as the mean (± SD) values from three assays and are expressed relative to the luciferase activity of the empty vector transfectant, which was set to 1.0.

E217K, Y95C, and wild-type PPARγ2 vectors and the empty vector were transfected into the 3T3-L1 preadipocyte cell line (ATCC, Manassas, VA, USA) using Lipofectamine 3000 (L3000008; Invitrogen); the cells were cultured in DMEM containing 25 mM glucose and 10% calf serum. The transfected 3T3-L1 preadipocytes were verified by RT-PCR and Western blotting (**ESM Figure 3**). Total RNA was extracted from cells using the Universal RNA Extraction Kit (9767; Takara, Kusatsu, Japan) and cDNA was synthesized using the PrimeScript RT Reagent Kit with gDNA Eraser (RR047A; Takara). Gene-specific primer sequences were used for RT-PCR (human PPARγ primer, 5′-AGCAAACCCCTATTCCATGCT-3′ and 5′-CACGGAGCTGATCCCAAAGT-3′; mouse β-actin primer, 5′-CACTGTCGAGTCGCGTCCA-3′ and 5′-GACCCATTCCCACCATCACA-3′). Proteins were collected from cells treated with RIPA Lysis Buffer (Applygen, C1053, Beijing, China) and were separated by SDS-PAGE and blotted on a nitrocellulose membrane. Differentiation and lipid droplet staining in transfected 3T3-L1 preadipocytes were evaluated on 24-well plates using the Adipogenesis Assay Kit (10006908; Cayman, Ann Arbor, MI, USA) according to the protocol of the manufacturer. The proteins of interest were detected by using antibodies against PPARγ (2435; Cell Signaling, Danvers, MA, USA) and β-actin (4970; Cell Signaling).

### Analysis of PPARG-DM

The pathogenicity of rare variants of *PPARγ2* was evaluated in accordance with the standards and guidelines recommended by the American College of Medical Genetics and Genomics (ACMG) (18) based on five lines of computational evidence [PROVEAN and SIFT (http://provean.jcvi.org), PolyPhen-2 (http://genetics.bwh.harvard.edu/pph2/index.shtml), MutationTaster (http://www.mutationtaster.org), and CADD (http://cadd.gs.washington.edu)].

### Screening for Hotspot Variants in the Expanded Samples (Cohort 2)

To estimate the frequency of a newly identified hotspot variant in *PPARγ2* in the general Chinese population, all patients (*n* = 545) with clinically diagnosed T2DM from a population-based cohort (Pinggu cohort) consisting of 3,345 individuals were screened. This cohort has been described in detail previously (17). To estimate the prevalence of diabetes induced by the hotspot variant in a hospital-based population, 1,200 more patients with diabetes diagnosed at ages older than 40 years (LOD) at the Peking University People’s Hospital from 2008 to 2017 were included. Additionally, 1,243 individuals older than 40 years with NGT and HbA_1c <_6% from the Pinggu cohort were screened for the hotspot variant as controls. Cohort 2 includes three parts of subjects: all patients with T2DM and all subjects older than 40 years with NGT from the Pinggu cohort and patients with LOD from a hospital-based population.

### Review of the Characteristics of Patients With Rare *PPARγ*2 Variants

A literature search was performed using PubMed, ClinVar, and the Human Gene Mutation Database for relevant *PPARγ* variants and their phenotypes (as of November 2019). The search terms were “PPARG,” “PPARgamma,” “*PPARγ*,” “Peroxisome proliferator-activated receptor γ,” “mutation,” “variant,” “lipoatrophy,” “lipodystrophy,” and “diabetes.” All articles published in English reporting patients with rare *PPARγ* alleles (frequency < 0.005) were included. The clinical features of patients with rare *PPARγ* alleles were evaluated.

### Statistical Analysis

All statistical tests were performed using SPSS version 22.0 for Mac (Chicago, IL, USA). Continuous variables are presented as the means and standard deviations (± SD) for normally distributed data or medians (25% and 75% quartiles) for the non-normally distributed data. Categorical variables are presented as numbers and percentages. When fewer than five data points were obtained, ranges are presented. Chi-square tests, Fisher’s exact tests, Student’s *t*-tests, and Mann–Whitney tests were used to compare the clinical characteristics between cases and controls.

The results of all functional experiments are expressed as the mean ± SD from three independent assays performed in duplicate. Differences were assessed by the Student’s *t*-test; differences with *P*-values <0.05 were considered statistically significant.

## Results

### Screening and Functional Analysis of Rare *PPARγ*2 Variants in Patients With EOD

In 1,002 patients with EOD, five rare variants [p.Val48Met(AF1), p.Glu217Lys(HD), p.Ser186Gly(DBD), p.Ile264Thr(HD), and p.Tyr95Cys(AF1)] were identified (**Table 1**). p.Glu217Lys was probably deleterious (19) and p.Val48Met was likely benign, according to a previous *in vitro* study (19), and the predictions were based on three or more lines of computational tools used in this study. The functions of the other three variants predicted as deleterious have not been reported previously.

**Table 1:** Rare variants in *PPARγ* gene detected in this study.

Position	Exons	Base change	AA change	Rs number^a^	ACMG^b^	MAF(%)^c^				
						ExAc	ExAc-East Asian	ExAc-European	ExAc-Africa	ExAc-Latino
12447410	Exon5	c.649G>A	E217K	rs766913119	Pathogenic	8E-6	NA	NA	NA	9E-5
12447552	Exon5	c.791T>C	I264T	NA	Likely pathogenic	NA	NA	NA	NA	NA
12421404	Exon2	c.284A>G	Y95C	rs1477623791	Likely pathogenic	NA	NA	NA	NA	NA
12434188	Exon4	c.556A>G	S186G	rs1421126930	Pathogenic	NA	NA	NA	NA	NA
12421262	Exon2	c.141G>A	V48M	rs141797536	Likely benign	8E-6	NA	1E-5	NA	NA

RefSeq: NM_015869.4, NP_056953.2.

a NA means this variant does not have an rs number.

b Classifying the rare variants identified in this study according to the standards and guidelines recommended by the American College of Medical Genetics (ACMG), detailed in **Supplementary Table 3**.

c NA indicates this variant does not have a minor allele frequency (MAF) in the ExAc-Global or ExAc-East Asian database; the MAF of this study indicates MAF in controls with normal glucose tolerance screened in this study; Nd indicates this mutation was not screened in controls with normal glucose tolerance.

In two pedigrees, the co-segregation of rare alleles and diabetes was evaluated. We observed that the homozygotic twin brother of the proband carrying p.Glu217Lys and a sister of the proband with p.Tyr95Cys all had diabetes and carried the same variants detected in the probands (**ESM Table 2** **and** **ESM Figure 4**).

**Figure 2:**
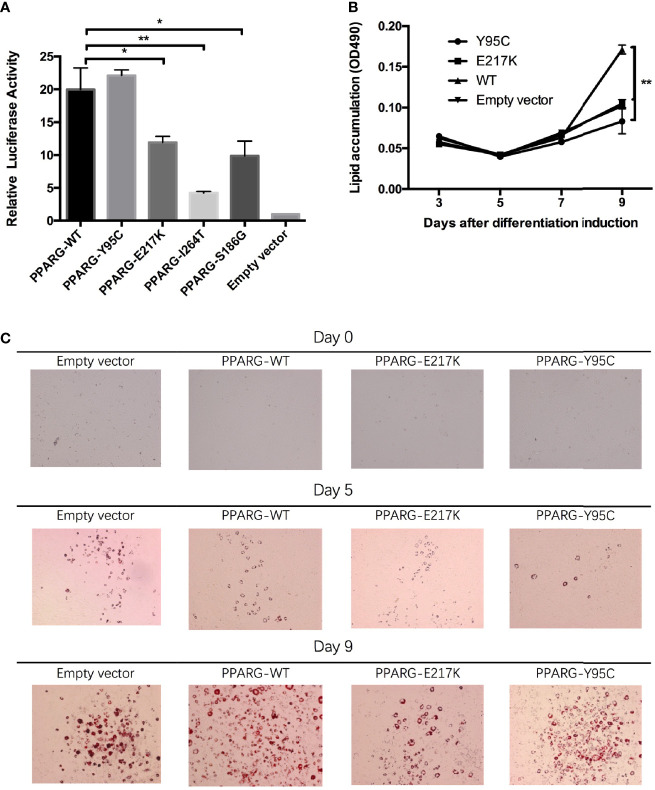
Functional analyses of constitutive mutant PPARγ2. Panel **(A)**, results of reporter gene assays, 293T cells were transfected with the reporter gene and wild-type *PPARγ2*, Y95C, I264T, S186G, and E217K expression vectors or the empty vector as described in the *Materials and Methods*. Compared with wild-type *PPARγ2* vector, both mutant vectors except Y95C-mutant vector show decreased relative transcription activity. Panels **(B, C)**, NIH 3T3-L1 preadipocyte transfected with the wild-type *PPARγ2* and two mutant *PPARγ2* (E217K and Y95C) genes were differentiated as described in the *Materials and Methods* section. The cells stained with Oil Red-O at indicated time points **(C)** and Oil Red-O were extracted and measured by spectrometry to identify lipid accumulation (**B**). Images at ×100 magnification. As compared with the wild-type *PPARγ2*, Y95C and E217K mutants decelerated the accumulation of lipid during differentiation. *P < 0.05, **P < 0.01.

In the *in vitro* assay, reporter gene activity levels in 293T cells expressing p.Glu217Lys (*P* = 0.015), p.Ile264Thr (*P* = 0.001), and p.Ser186Gly (*P* = 0.012) *PPARγ2* were significantly lower than those in cells expressing wild-type *PPARγ2* (**Figure 2A**). However, in cells transfected with the p.Tyr95Cys vector, reporter gene activity did not differ significantly (*P* = 0.334) from that of cells transfected with the wild-type vector.

As AF1 variants may influence *PPARγ2* expression *via* interactions with co-factors *in vivo* (5), we performed an adipocyte differentiation assay of the p.Tyr95Cys mutant. RT-PCR (**ESM Figure 3A**) and Western blotting (**ESM Figure 3B**) showed that the vectors containing p.Tyr95Cys, p.Glu217Lys, and wild-type *PPARγ2* were successfully transfected into 3T3 cells. As determined by Oil Red-O staining, less lipid accumulation was detected in the 3T3-L1 cells with the p.Tyr95Cys variant and in the positive control samples (p.Glu217Lys) than in cells with wild-type *PPARγ2* on day 9 of differentiation (**Figures 2B, C**, **ESM Table 3**).

Based on the ACMG classification system, experimental assays, or co-segregation analyses, we concluded that p.Ile264Thr, p.Tyr95Cys, and p.Ser186Gly are also likely pathogenic (**ESM Table 4**). Thus, six patients with PPARG-DM (0.6%) (three with p.Ile264Thr or p.Glu217Lys or p.Ser186Gly and three with Tyr95Cys; **ESM Figure 5**) were identified among 1,002 patients with EOD; 0.3% of these patients carried p.Tyr95Cys.

### Further Screening for p.Tyr95Cys in Expanded Samples (Cohort 2) to Estimate the Allele Frequency

Of 545 patients in the Pinggu cohort, one individual with diabetes carried p.Tyr95Cys. Thus, the estimated prevalence of p.Tyr95Cys-induced diabetes was 0.18% (1/545) in the population of diabetes patients, with an allele frequency of 0.03% (1/3,345) in the whole population.

Three subjects with p.Tyr95Cys were identified in the LOD cohort (*n* = 1,200), and the p.Tyr95Cys variant was not found among 1,243 individuals with NGT. Thus, the prevalence rates of p.Tyr95Cys-induced diabetes were about 0.3% (3/1002) and 0.25% (3/1200) in patients with EOD and LOD in a hospital-based population, respectively.

### Clinical Characteristics of Patients With PPARG-DM

The clinical features of 10 probands with PPARG-DM identified in this study (namely, seven with AF1 variants and three with non-AF1 variants) are shown in **Table 2**. Since lipodystrophy is associated with low leptin and adiponectin levels, serum leptin and adiponectin levels were also measured. In total, 802 of 1,002 patients with EOD for whom leptin and adiponectin levels were available were included in the analysis of the clinical features (**Table 3**). Only one patient with a variant in AF1 had a low leptin level, as assessed based on our normal reference range. The clinical phenotypes of patients included hyperinsulinemia, hypertriglyceridemia, low HDL-c levels, obesity, central obesity, DKD, and hypertension without a clear clustering pattern. Compared with patients with non-PPARG-DM in EOD, patients with PPARG-DM had higher UACR levels and there was a higher proportion of individuals with DKD (**Table 3**). As determined by Fisher’s test, PPARG-DM was associated with DKD (odds ratio of 12.5, 95% confidence interval 1.45–107.68, *P* = 0.009). There were no significant differences in the leptin and adiponectin levels between these two groups.

**Table 2:** Characteristics of patients with rare PPARγ variants detected in this study.

Individual no.	01-01	02-01	03-01	04-01	05-01	06-01	07-01	08-01	09-01	10-01
Variants	E217K	I264T	S186G	Y95C	Y95C	Y95C	Y95C	Y95C	Y95C	Y95C
Sex	Male	Male	Female	Male	Female	Female	Female	Male	Female	Male
Age at examination, years	34	45	28	26	21	38	65	70	60	62
Duration, years	1	5	2	3	0.1	0.6	20	11	1	0.1
Waist circumference, cm	79	97	70	116	80	85	108	89	81	84
BMI, kg/m^2^	25	27.4	23.0	32.6	20.7	25.7	30.8	22.23	27.77	25.1
SBP, mmHg	120	Nd	110	140	93	120	120	130	122	130
DBP, mmHg	70	Nd	90	90	64	85	70	70	80	80
Pancreatitis	No	No	No	No	No	No	No	Nd	Nd	No
Hypertension	No	No	No	No	No	No	Yes	Yes	Yes	No
Stroke	No	No	No	No	No	No	No	No	Yes	No
Coronary heart disease	No	No	No	No	No	No	No	No	No	No
NAFLD	Yes	Nd	No	Yes	No	No	Yes	Nd	Nd	Nd
Diabetic retinopathy	No	Nd	No	No	Yes	No	No	No	No	No
OHA	Metformin glimepiride	Metformin	Metformin glimepiride	Metformin	Metformin	Metformin voglibose	Metformin acarbose	Yes	Yes	No
Insulin therapy	No	No	No	Yes	No	No	No	No	No	No
FBG, mmol/L	13.61	12.5	7.3	7.94	7.22	4.87	6.55	6.77	7.01	7.18
Fins, μU/ml	44.2	7.8	20.7	12.1	5.94	Nd	8.43	7.68	17.96	2.66
HbA_1c_, %(mmol/mol)	10.4 (90.1)	10.6 (92.3)	6.5 (47.5)	6.9 (51.9)	10.4 (90.1)	8.5 (69.4)	6.3 (45.3)	5.8 (39.9)	5.5 (36.6)	5.8 (39.9)
HDL-c, mmol/L	0.7	1.0	0.93	0.9	0.79	1.03	1.12	0.86	1.69	1.38
LDL-c, mmol/L	2.5	2.7	3.1	2.4	1.69	2.54	2.46	2.47	4.51	2.33
TCHO, mmol/L	4.7	5.4	4.7	3.7	3.44	4.42	5.14	3.71	6.3	4.15
Triglyceride, mmol/L	9.4	3.3	2.5	1.7	1.23	1.42	2.71	1.46	1.03	0.57
UA, μmol/L	301	Nd	414	498	437	283	476	Nd	Nd	201
CRE, μmol/L	73	Nd	62	48	48	49	64	78.6	53.9	71
hs-CRP, mg/L	Nd	Nd	0.87	1.82	0.25	Nd	1.99	Nd	Nd	Nd
eGFR, ml/min/1.73 m^2^	93.1	Nd	118	163.9	170.3	149.3	97.6	94.6	122.3	109.6
UACR, mg/g	1813.6	53.1	431	257.8	35.49	5mg/L (urine albumin)	7.65	Nd	Nd	1.24
Adiponectin, μg/ml	0.99	0.39	1.56	4.74	4.38	1.48	2.884	Nd	Nd	12.162
Leptin, ng/ml	1.02	0.98	8.47	14.15	1.91	6.5	32.434	Nd	Nd	5.054

Nd, not determined; NA, not applicable; Age, age at examination; BMI, body mass index; OHA, oral hypoglycemic agent; SBP, systolic blood pressure; DBP, diastolic blood pressure; NAFLD: non-alcoholic fatty liver disease; FPG, fasting plasma glucose; HbA_1c_, hemoglobin A1c; Fins, fasting serum insulin; UA, serum uric acid; TCHO, total cholesterol, LDL-c, low-density lipoprotein cholesterol; HDL-c, high-density lipoprotein cholesterol; CRE, serum creatinine; hs-CRP, high sensitivity C-reactive protein; UACR, urinary albumin/creatinine ratio.

**Table 3:** Characteristics of PPARG-DM and non-PPARG-DM patients.

Items	PPARG-DM (*n* = 6)	Non-PPARG-DM (*n* = 796)	*P*-value
Sex, female/male	3/3	247/549	0.383
Age, years	31 (25, 40)	34 ± 9	0.608
Waist circumference, cm			
Male	79–116^a^	96 (88, 103)	0.881
Female	80–85^a^	88 (80, 98)	0.336
BMI, kg/m^2^	23.2 (20.9, 28.7)	27.1 ± 4.7	0.163
SBP, mmHg	115 (106, 125)	126 ± 17	0.146
DBP, mmHg	80 (67, 88)	79 ± 12	0.869
Hypertension, *n* (%)	1 (17)	161 (20)	1
Dyslipidemia, *n* (%)	5 (83)	685 (86)	0.596
Coronary heart disease, *n* (%)	1 (17)	18 (2)	0.134
Stroke, *n* (%)	0 (0)	17 (2)	1
DKD, *n* (%)	5 (83)	196 (29)^b^	0.009
DR, *n* (%)	1 (17)	93 (12)	0.528
FPG, mmol/L	7.6 (6.6, 12.8)	8.9 ± 3.5	0.912
Fins, μU/ml	12.1 (6.9, 32.5)	11.6 (7.2, 17.9)	0.704
HbA_1c_, %	9.5 (6.8, 10.5)	9.2 ± 2.5	0.834
HbA_1c_, mmol/mol	79.8 (50.8, 90.7)	77.5 ± 26.9	0.834
ALT, U/L	11–61	25 (16.46)	0.763
AST, U/L	12–29	20 (16.29)	0.339
HDL-c, mmol/L			
Male	0.70–0.98^a^	1.00 ± 0.22	0.291
Female	0.79–0.93^a^	1.15 ± 0.35	0.098
TCHO, mmol/L	4.6 (3.7, 4.9)	4.88 ± 1.32	0.326
LDL-c, mmol/L	2.5 (2.2, 2.8)	2.94 ± 0.90	0.154
Triglyceride, mmol/L	2.1 (1.4, 4.8)	1.67 (1.14, 2.75)	0.35
UA, μmol/L	414 (292, 468)	359 ± 103	0.48
CRE, µmol/L	49 (48, 68)	62 (51, 72)	0.31
eGFR, ml/min/1.73 m^2^	149 (106, 167)	144 ± 62	0.956
Hs-CRP, mg/L	0.25–1.82^a^	1.88 (0.88, 4.02)	0.148
UACR, mg/g	257.8 (44.3, 1,122.3)	9.0 (4.2, 35.8)	0.004
HOMA-IR	4.4 (3.1, 16.7)	4.1 (2.5, 7.4)	0.555
HOMA-β	54.4 (24.6, 98.2)	54.6 (27.7, 104.6)	0.836
Adiponectin, μg/ml			
Male	0.39–4.74^a^	1.42 (0.74, 2.82)	0.774
Female	1.48–4.38^a^	1.70 (0.90, 3.52)	0.658
Leptin, ng/ml			
Male	0.98–14.15^a^	5.28 (2.99, 8.92)	0.333
Female	1.91–8.47^a^	12.58 (6.46, 18.83)	0.072

The PPARG-DM group only had six subjects, the distribution of all continuous variables in this group was non-normal, P-values were obtained by non-parametric test (Mann–Whitney test), and Fisher’s test was used to compare the categorical variables. Continuous variables are presented as means and standard deviations (± SD) of the normally distributed data or as medians (25% and 75% quartiles) for the non-normally distributed data.

Age, age at examination; BMI, body mass index; SBP, systolic blood pressure; DBP, diastolic blood pressure; DKD, diabetic kidney disease; DR, diabetic retinopathy; FPG, fasting plasma glucose; HbA_1c_, hemoglobin A1c; Fins, fasting serum insulin; UA, serum uric acid; ALT, Alanine transaminase; AST, Aspartate transaminase; TCHO, total cholesterol, LDL-c, low-density lipoprotein cholesterol; HDL-c, high-density lipoprotein cholesterol; CRE, serum creatinine; hs-CRP, high sensitivity C-reaction protein, UACR, urinary albumin/creatinine ratio.

a The number of valid data was less than 5; data presented as range.

b One hundred ten patients who did not test for UACR in the non-PPARG-DM group, 686 patients in the non-PPARG-DM group, and 6 patients in the PPARG-DM group were included to compare the prevalence of DKD between two groups.

We compared the clinical features of patients with non-AF1 and AF1 variants. All patients with non-AF1 variants had hypertriglyceridemia, DKD, and a BMI of less than 28 kg/m^2^. For patients with p.Tyr95Cys in AF1, the BMI values ranged from 20.7 to 32.6 kg/m^2^, and two of seven patients had hypertriglyceridemia. Compared with patients carrying non-AF1 variants (*n* = 4, including one relative with PPARG-DM), patients carrying AF1 variants (*n* = 8, including one relative with PPARG-DM) had higher adiponectin levels (median: 0.69 *vs*. 4.38 µg/ml, *P* = 0.03, data not shown).

To determine the effect of thiazolidinediones on patients with PPARG-DM, we followed the proband with p.Glu217Lys for 1 year and observed the response to pioglitazone. Before the patient was diagnosed with PPARG-DM, he had been taking glimepiride (4 mg/day) and metformin (1,500 mg/day) and had occasional hypoglycemia. After glimepiride was replaced with pioglitazone (30 mg/day) for 3 months, his glucose control did not change but his triglyceride, insulin, and inflammatory marker levels decreased (**ESM Table 5**), with no hypoglycemia or reduced eGFR.

### Summary of Clinical Features of Patients With Rare *PPARγ*2 Alleles Reported in Previous Studies

**Figure 3:**
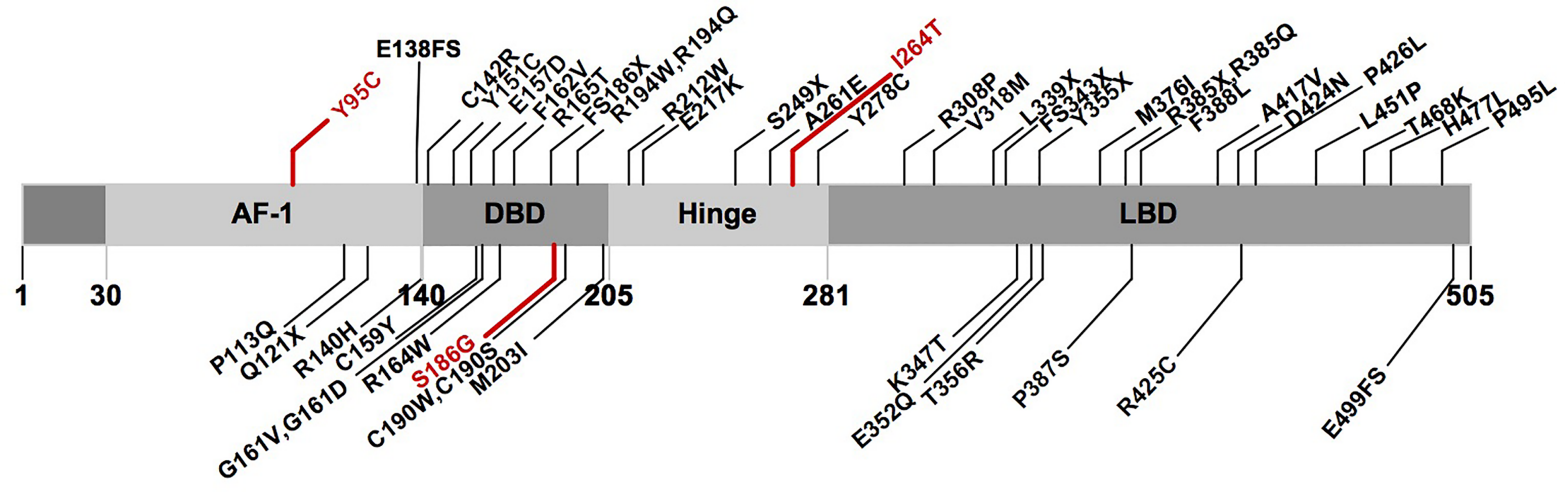
Schematic representation of the PPARγ2 domain structure and position of the rare variants identified in previous studies and this study. Marked in red: novel variants described in this study. AF1, activation function 1 domain; DBD, DNA-binding domain; LBD, ligand-binding domain.

A total of 68 probands with 50 rare *PPARγ*2 variants have been reported in the literature, namely, 6 with nonsense variants, 4 with frameshift variants, 1 with a compound heterozygous variant, and 57 with heterozygous variants (**ESM Table 6**). In total, 46 rare variants (3 in AF1, 16 in DBD, 5 in HD, and 22 in LBD) from 64 probands were classified as pathogenic or likely pathogenic according to the ACMG (**ESM Table 7** and **Figure 3**).

Per the available clinical data for probands with likely pathogenic *PPARγ2* variants, 89% (57/64) were diagnosed with diabetes or impaired glucose tolerance, 86% (32/37) were diagnosed before 40 years of age, and 85% patients (11/13) showed an improvement in glucose control after thiazolidinedione therapy. Among six probands for whom information regarding diabetic complications was available, five (83%) had DKD. Additionally, 93% (52/56) of the probands had hypertriglyceridemia, 36% (4/11) had recurrent pancreatitis, and 96% (27/28) had hepatic steatosis. Among 14 patients for whom relevant data were available, four (29%) had low leptin levels (**ESM Table 6**).

Compared with the probands (*n* = 57) with rare pathogenic variants in the non-AF1 domains of *PPARγ2*, the probands (*n* = 7) with rare pathogenic variants in AF1 had a lower prevalence of lipodystrophy (1/7), higher frequency of obesity (5/7), and relatively normal serum triglyceride levels; the triglyceride levels were only evaluated in two patients, one of whom showed an average value (over 30 years) of 0.14 mmol/L and the other showed a value of 1.7 mmol/L (**Table 4**).

**Table 4:** The summary of clinical features of subjects with rare PPARγ variants from previous studies.

Domain	Cases	BMI	Overweight	Obesity	Lipoatrophy^*^	Diabetes^a^	Hepatic steatosis	Fat whole body, %^b^	HDL-c, mmol/L		Triglycerides, mmol/L	HbA_1c_, % (mmol/mol)	Fasting insulin, μU/ml
									Male	Female			
Only probands													
AF1	3F, 3M, 1UC	38 (29, 44)	6/7	5/7	1/7	4/7 (1 IGT)	0/0	Nd	Nd	1.10–1.68	0.14–1.7	Nd	8.0–28.5
DBD	17F, 5M, 1UC^c^	26 ± 4	12/21	5/21	14/23	21/23 (1 IGT)	10/10	22–33^d^	1.19 (0.85, 1.61)	0.78 ± 0.27	5.5 (2.5, 23.4)	7.4 ± 1.7 (59.7 ± 22.0)	27.2 (21.8, 99.6)
HD	6F	26 (21, 27)	4/6	0/6	1/6	6/6	1/1	Nd	Nd	0.81 ± 0.36	2.5 (2.3, 12.6)	5.6–9.2 (37.7–77.0)	44.1–180.4
LBD	24F, 3M, 1UC	27 ± 4	16/26	6/26	22/28	24/28	15/17	20 (16, 25)^e^	0.73–1.48	0.83 ± 0.25	4.7 (2.5, 12.5)	7.6 ± 1.8 (56.7 ± 17.8)	24.5 (18.6, 58.3)
Non-AF1	47F, 8M, 2UC	26 ± 4	32/53	11/53	37/57	51/57 (1 IGT)	26/28	22 (19, 26)	1.17 (0.81, 1.45)	0.81 ± 0.27	4.9 (2.4, 11.8)	7.5 ± 1.7 (57.7 ± 19.0)	26.8 (21.4, 60.8)
*P*-value^f^ (AF1 *vs*. non-AF1)		0.013	0.246	0.012	0.015	0.166	Nd	Nd	Nd	Nd	Nd	Nd	Nd

Continuous variables are presented as means and standard deviations (± SD) of the normally distributed data or as medians (25% and 75% quartiles) for the non-normally distributed data. If observed value <5, data would be presented as range. “xx/xx” represents the prevalence of the disease: the numerator indicates the number of cases with the disease, and the denominator represents the total number of cases with data provided by literature. Overweight indicates BMI >25 kg/m^2^; obesity indicates BMI >30 kg/m^2^.

“Nd” indicates data missing or data was not analyzed; “UC” indicates sex is uncertain; F, female; M, male; BMI, body mass index; HbA_1c_, hemoglobin A1c; LDL-c, low-density lipoprotein cholesterol; HDL-c, high-density lipoprotein cholesterol; AF1, activation function 1 domain; DBD, DNA-binding domain; HD, hinge domain; LBD, ligand-binding domain. SI/non-SI conversion calculated the following: insulin, pmol/L = µU/ml * 6.945; HDL, mmol/L * 38.61 = mg/dl; LDL, mmol/L * 38.61 = mg/dl; TG, mmol/L * 88.50 = mg/dl.

a Because lipoatrophy and diabetes were not missed generally, all cases that did not provide data were considered to be not with lipoatrophy or diabetes.

b Fat content was measured by dual-energy X-ray absorptiometry (DEXA).

c A family with E157D variant consists of seven females and eight males; means or medians were used in analysis.

d A total of four patients had measured total body fat by DEXA.

e A total of eight patients had measured total body fat by DEXA.

f The non-parametric test was used to analyze BMI. The chi-square test was used to analyze categorical variables. Patient with IGT was classified in the case group in chi-square test. Fisher’s exact test was used to analyze overweight, obesity, lipoatrophy, and diabetes variables.

## Discussion

In this study, we obtained the following novel findings: 1) we obtained the first estimate of the prevalence of PPARG-DM in a Chinese population with EOD, i.e., 0.6%. 2) A hotspot diabetes-related variant (p.Tyr95Cys) was identified in AF1; this variant suppressed the differentiation of 3T3-L1 preadipocytes *in vitro*. 3) By a review of all reported cases with rare damaging *PPARγ*2 variants, we found distinct phenotype differences between patients with AF1 and non-AF1 variants in *PPARγ2*, as confirmed in the patients evaluated in this study. 4) Most patients with PPARG-DM had DKD, supporting previous findings that PPARγ is involved in the development of DKD, based on animal studies and meta-analyses of studies on patients with diabetes.

Therefore, patients with EOD and metabolic syndrome should be screened for *PPARγ* variants, even if they lack typical lipodystrophy features. These patients with pathogenic *PPARγ* variants have a higher risk of DKD, and early genetic diagnosis is helpful to improve the clinical outcomes of these patients.

Almost all previously reported cases with rare variants were from Caucasian or Latino populations (**ESM Table 6**). Only one study has reported the prevalence of rare variants of *PPARγ2* in East Asians (19) by the exon sequencing of nearly 20,000 individuals (9,070 patients with T2DM and 10,682 controls) from multiethnic populations; in this previous study, 0.1% of individuals with T2DM carried functional rare variants of *PPARγ2*. Of note, only 998 patients with T2DM and 1,146 controls from East Asian populations (Korea and Singapore) were included in this study, and it is not clear whether the rare pathogenic variants of *PPARγ2* existed in East Asian subpopulations. The prevalence of T2DM in China is as high as 11.6% (20); accordingly, a larger sample size is needed to estimate the prevalence of PPARG-DM in East Asians to enable precision medicine. Because we found that most reported patients with PPARG-DM were diagnosed before the age of 40, we estimated the prevalence of PPARG-DM in this population to ensure that our results are clinically meaningful. We sequenced *PPARγ2* in 1,002 Chinese patients with EOD and identified 6 patients (0.6%) with PPARG-DM, confirming the observations that PPARG-DM is more common in patients with EOD than in those with LOD. Because patients with EOD account for approximately 16.2% of all Chinese patients with T2DM (20), the estimated prevalence of PPARG-DM in patients with T2DM in China was approximately 0.1%. Thus, we concluded that the prevalence of PPARG-DM is similar in Caucasian and Chinese populations, and the smaller number of reported cases of PPARG-DM in China may be explained by the relatively low awareness of PPARG-DM among physicians in China and a lack of cost-effective genetic tests. In this study, maturity-onset diabetes of the young (MODY) was not ruled out; however, monogenic diabetes accounts for about 1%–5% of all cases of diabetes in young people. Moreover, PPARG-DM is characterized by insulin resistance, which is not consistent with the phenotype of MODY. Accordingly, we expect MODY to have a negligible impact on the results of the present study.

We identified the p.Tyr95Cys variant in AF1 in *PPARγ2* as a novel rare variant. This was the most frequent variant in patients with PPARG-DM (3/6) in the EOD cohort; in a hospital-based population, about 0.3% of patients with EOD and 0.25% patients with LOD carried this variant. The frequency of p.Tyr95Cys in the Chinese population is 0.009% (1/10,588) (http://www.mbiobank.com/), which is significantly lower than that in patients with EOD (*P* = 0.002, data not shown). We subsequently screened for p.Tyr95Cys in a randomly sampled cohort (Pinggu cohort) and found a p.Tyr95Cys frequency of 0.18% in patients with diabetes. As reported, several population-specific rare or low-frequency variants (p.Gly319Ser in *HNF1A* in the Oji-Cree population, p.Glu508Lys in *HNF1A* in a Latino population, and p.Arg1420His in *ABCC8* in a Southwest American Indian population) (21–23) are associated with an increased risk for diabetes with a large effect size. No individual was found to carry the p.Tyr95Cys variant in online databases, such as ExAC and 1000 Genomes. Thus, p.Tyr95Cys may be a Chinese-specific causative variant for diabetes.

The clinical characteristics and pathogenesis of variants in the PPARγ AF1 domain are still unclear. As summarized in **Table 4**, patients with rare variants in AF1 had milder insulin resistance, greater fat mass, and lower triglyceride levels than patients with rare variants in non-AF1 domains. These clinical features were similar to those of patients with p.Tyr95Cys (**Table 2**) in this study. The AF1 domain of PPARγ2 is involved in the transactivation and recognition of the protein by transcriptional coactivators and affects the ligand-binding affinity of the LBD (24). Previous studies have demonstrated that posttranslational modifications in AF1, including phosphorylation (25, 26), acetylation (27), *O*-GlcNAcylation (28), and SUMOylation (1, 29), regulate the functions of PPARγ *in vitro*. A variant in an important phosphorylation site, Ser112, of PPARγ2 (p.Ser112Ala) has been found to protect mice from obesity-induced insulin resistance (30). Subjects with the rare Pro113Gln variant near Ser112 show increased adipocyte differentiation *via* Ser112 phosphorylation. p.Pro113Gln can cause a range of metabolic symptoms, including obesity, T2DM, and high fasting insulin levels (7, 31). These phenotypic differences among patients might be partially explained by the interaction of PPARγ with co-factors or environmental factors. Further research is necessary to unravel the complex relationship between genotypes, posttranslational modification status, environmental factors, and disease risk. Differences between the effects of variants in AF1 and other domains may provide alternative therapeutic strategies to thiazolidinedione.

There is a relationship between the SUMOylation and phosphorylation of PPARγ2. The Ser112Ala variant reduces the SUMOylation of PPARγ2, while the phospho-mimicking Ser112Asp variant increases the SUMOylation of Lys107 (32). The FGF21–PPARγ pathway is a self-reinforcing regulatory loop (33). The SUMOylation of Lys107 is regulated by FGF21 *in vivo* (33). PPARγ2 is expressed in both adipocytes and hepatocytes. The development of fatty livers in many mouse models of obesity and diabetes is associated with the increased expression of PPARγ2 (4). FGF21 is more abundant in the liver than in adipose tissues, explaining why some patients with p.Tyr95Cys have hyperglycemia but mild hypertriglyceridemia and lipoatrophy. In addition, as reported, Lys94 is potentially modified by SUMOylation (1). Accordingly, the p.Tyr95Cys variant, in addition to affecting phosphorylation, may suppress the differentiation of adipocytes by influencing SUMOylation. Further studies are needed to verify whether p.Tyr95Cys alters phosphorylation; in particular, future studies should include a negative control transfected with a Ser112Ala mutant. Additionally, immunoprecipitation assays are needed to determine whether p.Tyr95Cys affects the SUMOylation of PPARγ.

Patients with lipodystrophy usually show low leptin levels; in our review, only 14 reported patients harboring *PPARγ2* variants were evaluated for leptin levels, 4 of which had low leptin levels. In our study, leptin levels were measured in 10 patients with PPARG-DM (3 with variants in non-AF1 domains and 7 with the p.Tyr95Cys variant); no differences were observed between PPARG-DM and T2DM, and only 1 patient with p.Tyr95Cys had low leptin levels (**Table 2**). These observations indicate that the serum leptin cutoff value derived from the normal reference range was not helpful for distinguishing PPARG-DM from T2DM. In fact, the pathogenesis of PPARG-DM may be highly complex because more organs (e.g., the liver, adipocyte, brain, and islet) are involved in its development (34, 35). Thus, leptin levels have limited value for PPARG-DM screening in adults.

About two decades ago, p.Pro12Ala in PPARγ2 was found to be associated with T2DM in a candidate gene study, and this was confirmed in a subsequent genome-wide association study (GWAS) (36). However, this association was not replicated in a later GWAS (37) and meta-analysis (38) of the Chinese population. Similarly, this variant was recently reported to be associated with DKD in Caucasians; however, a similar association was not detected in Asians (8); this may be explained by the low frequency of Ala12 and the low effect size of this variant in Asians. Recently, an exome sequencing study has shown that the risk of diabetes was 7.22-fold higher in carriers of rare variants with reduced PPARγ function (19). However, renal phenotypes have rarely been described in subjects with PPARG-DM. Our results showed that the risk of DKD was 12.5-fold higher in patients with PPARG-DM in the EOD cohort. However, the mechanism underlying the effects of these mutations in PPARγ on DKD is not clear. Bonofiglio et al. (39) have found that PPARγ may be involved in the regulation of phosphatase and tensin homolog deleted on chromosome 10 (PTEN) expression by binding to the peroxisomal proliferator response element (PPRE) upstream of the PTEN promoter. PTEN plays a significant role in DKD. Previous studies have found that PTEN is involved in the regulation of the epithelial-to-mesenchymal transition (EMT) in renal tubular cells, tubulointerstitial fibrosis (40), and cytoskeletal rearrangement in podocytes (41). Accordingly, PPARγ may participate in the development of DKD by regulating PTEN expression. Further studies of these variants and the expression of PTEN are needed.

Among the reported PPARG-DM patients taking thiazolidinediones, most showed improved glycemic control (**ESM Table 6**). The lack of response to thiazolidinediones may be due to the nearly complete loss-of-function mutations in PPARγ (19). In the present study, we also found that a patient with a partial inactivation of PPARγ had a better response to thiazolidinedione, with improvements in glucose levels, lipid metabolism, and inflammatory indicators (**ESM Table 5**). Thiazolidinediones could inhibit the proliferation of cultured mesangial cells (42) and downregulate the expression of vascular endothelial growth factor (VEGF) by inhibiting the expression of hypoxia-inducible factor-1α (HIF-1α) in the kidneys of diabetic rats, thereby protecting the kidneys (43). Moreover, thiazolidinediones are associated with the attenuation of DKD in patients with T2DM (44, 45); the activation of PPARγ slows the progression of DKD, and several mechanisms, both systemic and renal, have been implicated in the renoprotective effects of PPARγ (46). Thus, thiazolidinediones may serve as a good therapeutic choice for PPARG-DM. It is possible that patients carrying the rare variants in AF1 have an incomplete loss of function of PPARγ and, therefore, show a response to thiazolidinediones.

This study had a few limitations. First, fat mass was not measured. Second, cell-based studies of the pathogenicity of the variants are limited; thus, more detailed functional studies are needed to validate this aspect. Third, the association of PPARγ with DKD is still not clear and should be evaluated further. Fourth, the prevalence of PPARG-DM in a population-based sample should be estimated by screening all exons of *PPARγ2*. Some patients with PPARG-DM were only screened for the *PPARγ* gene, whereas the possibility of other diabetes-inducing genetic variants cannot be excluded. Fifth, the sample size of patients with PPARG-DM was small and potential confounders could not be adjusted in comparative analyses; hence, further studies with a larger sample size are needed.

## Conclusions

The prevalence of PPARG-DM in the Chinese population is similar to that reported in other populations; PPARG-DM accounts for 0.6% of Chinese patients with EOD. The p.Tyr95Cys variant in AF1 is a novel hotspot variant in the Chinese population. Patients with PPARG-DM caused by AF1 variants often lack typical lipodystrophy features. Additionally, patients with PPARG-DM have an increased risk for DKD. The individualized application of thiazolidinedione may greatly benefit patients with PPARG-DM, especially those with DKD.

## Data Availability Statement

The datasets presented in this study can be found in online repositories. The names of the repository/repositories and accession number(s) can be found below: https://www.ncbi.nlm.nih.gov/ and rs141797536, rs1477623791, rs1421126930, and rs766913119.

## Ethics Statement

The studies involving human participants were reviewed and approved by the Ethics Committee of Peking University People’s Hospital. The patients/participants provided their written informed consent to participate in this study.

## Author Contributions

SG conducted the research, performed the literature review, collected the data, analyzed the data, and wrote the manuscript. ML, XC, WL, YYL, S-mZ, LZ, YM, XTH, YFL, XZ, YZ, QW, LC, and QR helped to collect the data and reviewed the manuscript. PZ helped to perform the experiments. LJ and XYH designed the study, contributed to the discussion, and reviewed and edited the manuscript. SG, XYH and LJ are the guarantors of this work and, as such, have full access to all the data reported in the study and take responsibility for the integrity of the data and the accuracy of the data analysis. All authors contributed to the article and approved the submitted version.

## Funding

This study was supported by grants from the National Key Research and Development Program of China (2016YFC1304901), Beijing Municipal Science and Technology Commission Funding (Z141100007414002 and D131100005313008), National High-Technology Research and Development Program of China (2012AA02A509), and National Natural Science Foundation of China (82000759).

## Conflict of Interest

The authors declare that the research was conducted in the absence of any commercial or financial relationships that could be construed as a potential conflict of interest.

## Publisher’s Note

All claims expressed in this article are solely those of the authors and do not necessarily represent those of their affiliated organizations, or those of the publisher, the editors and the reviewers. Any product that may be evaluated in this article, or claim that may be made by its manufacturer, is not guaranteed or endorsed by the publisher.

## Abbreviations

AF1, activation function 1 domain; ACMG, American College of Medical Genetics and Genomics; DKD, diabetic kidney disease; DBD, DNA-binding domain; EOD, early-onset type 2 diabetes; FPG, fasting plasma glucose; Fins, fasting insulin; GWAS, genome-wide association study; HD, hinge domain; LOD, late-onset type 2 diabetes; LBD, ligand-binding domain; NGT, normal glucose tolerance; PPARG-DM, *PPARγ* variant-induced diabetes; T2DM, type 2 diabetes.
