# Supplementary material for: Genetics and Clinical Characteristics of PPARγ Variant-Induced Diabetes in a Chinese Han Population
Source: Front Endocrinol (Lausanne). 2021 Oct 26;12:677130. doi: 10.3389/fendo.2021.677130 (PMC8576343; doi:10.3389/fendo.2021.677130)
Supplement: Supplementary file 2 [file DataSheet_2.docx]

**Supplementary Material**

**Supplementary Tables**

ESM Table 1. PPARG premiers sequences used in PCR.

| Primers | Sequences (5'to3') |
| --- | --- |
| PPARG-E1F | CCTCACAAGACACTGAACA |
| PPARG-E1R | AACACAACCTGGAAGACAA |
| PPARG-E2F | CTGTGAGATTGCTGTGTTC |
| PPARG-E2R | AATGAATGGTGAATGCCTTC |
| PPARG-E3F | CAGGTGAGGCTTAGAGATG |
| PPARG-E3R | GGTGACAGAGCAAGATACT |
| PPARG-E4F | AGAATCGTGTCAAGAACCT |
| PPARG-E4R | TGGCAATGGCTTTAGTGT |
| PPARG-E5F | CTGATGGTCTGTGCTACTT |
| PPARG-E5R | TGTTGACTTGTGCTATCTGA |
| PPARG-E6F | GCAGCCATTCGTTATCTATG |
| PPARG-E6R | CTTCACACCGCAAACCTA |
| PPARG-E7F | GATTCCATCCTTAGTTCTTACC |
| PPARG-E7R | CAGCAGTTCCACTCACATA |

ESM Table 2. Characteristics of family members with PPARG variants detected in this study.

| Individual No. | 01-02 | 07-02 |
| --- | --- | --- |
| Variants | E217K | Y95C |
| Sex | Male | Female |
| Age, yrs | 35 | 65 |
| Duration of diabetes, yrs | 7 | 2 |
| Waist circumference，cm | 85 | 76 |
| BMI, kg/m^2^ | 23.6 | 23.4 |
| SBP，mmHg | 103 | 142 |
| DBP，mmHg | 52 | 84 |
| Hypertension | No | Yes |
| Stroke | No | No |
| Coronary heart disease | No | No |
| Dyslipidemia | Yes | Yes |
| Diabetic retinopathy | No | No |
| OHA | Metformin, miglitol | Metformin |
| Insulin therapy | No | No |
| FPG，mmol/l | 5.32 | 8.48 |
| Fins，uU/ml | 5.78 | 7.85 |
| HbA1c，%(mmol/mol) | 7.7(60.6) | 6.2(44.2) |
| HDL-c,mmol/l | 0.84 | 1.46 |
| LDL-c，mmol/l | 2.76 | 2.18 |
| TCHO，mmol/l | 3.98 | 3.96 |
| Triglyceride，mmol/l | 1.34 | 1.64 |
| Uric acid，umol/l | 311 | 273 |
| CRE，umol/l | 65 | 68 |
| hs-CRP，mg/l | 1.29 | 3.78 |
| eGFR，ml/min/1.73m^2^ | 136 | 91 |
| UACR，mg/g | 208.5 | 5.26 |
| Adiponectin，ug/ml | 0.314 | / |
| Leptin，ng/ml | 1.017 | / |

Age, age at examination; BMI, body mass index; OHA, oral hypoglycemic agent; SBP, systolic blood pressure; DBP, diastolic blood pressure; FPG, fasting plasma glucose; HbA1c, hemoglobin A1c; Fins, fasting serum insulin; TCHO, total cholesterol, LDL-c, low-density lipoprotein cholesterol; HDL-c, high-density lipoprotein cholesterol; CRE, serum creatinine; hs-CRP, high sensitivity C-reaction protein, UACR, urinary albumin/creatinine ratio.

ESM Table 3. The differentiated cells stained with Oil Red O and the absorbance at OD490nm.

|  | Day3 | Day5 | Day7 | Day9 |
| --- | --- | --- | --- | --- |
| Empty vector | 0.058 | 0.041 | 0.069 | 0.102 |
| Y95C | 0.065 | 0.040 | 0.058 | 0.083 |
| E217K | 0.055 | 0.042 | 0.067 | 0.105 |
| Wild type | 0.062 | 0.042 | 0.063 | 0.171 |

Means of 3 independent assays.

ESM Table 4. Classifying the rare variants identified in this study according to the standards and guidelines recommended by the American College of Medical Genetics (ACMG)^30^

| Variants | Glu217Lys | Ile264Thr^a^ | Tyr95Cys^a^ | Ser186Gly^a^ | Val48Met |
| --- | --- | --- | --- | --- | --- |
| PVS1 |  |  |  |  |  |
| PS1 |  |  |  |  |  |
| PS2 |  |  |  |  |  |
| PS3 | √^b,21^ | √ | √ | √ |  |
| PS4 |  |  |  |  |  |
| PM1 |  |  |  | √ |  |
| PM2 | √ | √ | √ | √ |  |
| PM3 |  |  |  |  |  |
| PM4 |  |  |  |  |  |
| PM5 |  |  |  |  |  |
| PM6 |  |  |  |  |  |
| PP1 | √ |  | √ |  |  |
| PP2 | √ | √ | √ | √ | √ |
| PP3 | √ | √ | √ | √ |  |
| PP4 | √ | √ |  | √ |  |
| PP5 |  |  |  |  |  |
| Classification | Pathogenic | Likely Pathogenic | Likely Pathogenic | Pathogenic | Likely Benign^c^ |

a．Novel variant found in this study; b. evidence from the other studies. √ evidence from this study;

PM1 in this study: S186G located at a critical and well-established functional domain for DNA binding;

PP3 in this study: three or more lines of computational tools (PROVEAN, SIFT, Polyphen-2, Mutation Taster and CADD) predict that the variants are damaging or probably damaging.

Glu217Lys was located at the hinge domain. A functional study and this study revealed that this variant had a reduced effect on PPARG activity. This variant was predicted to be deleterious by multiple lines computational tools.

Ile264Thr was located at the hinge domain. This study revealed that this variant had a reduced effect on PPARG activity. This variant was predicted to be deleterious by multiple lines computational tools, and was not present in 1000G or ExAC.

Tyr95Cys was located at the AF1 domain. This study revealed that this variant had a reduced effect on PPARG activity. This variant was predicted to be deleterious by multiple lines computational tools, and was not present in 1000G or ExAC.

Ser186Gly was located at the DNA binding domain. This study revealed that this variant had a reduced effect on PPARG activity. The parents of the patient who carried this variant had not diabetes but not be confirmed by genetic test. This variant was predicted to be deleterious by multiple lines computational tools, and was not present in 1000G or ExAC.

c. Val48Met was benign according to the in vitro study^21^ and the prediction based on three or more lines of computational tools used in this study, evidence of benign impact are BS3 and BP4, so the variant could classified as “likely benign”.

ESM Table 5. Follow up of probands with E217K mutation of PPARG.

|  | Before treatment with pioglitazone^a^ | Pioglitazone 30mg Qd for 3months^b^ |
| --- | --- | --- |
| BMI, kg/m^2^ | 25 | 26 |
| FPG，mmol/l | 6.14 | 6.35 |
| FCP, ng/ml | 4.67 | 3.53 |
| Fins，uU/ml | 18.75 | 15.37 |
| HbA1c，%(mmol/mol) | 6.4(46.4) | 6.4(46.4) |
| HDL-c,mmol/l | 0.96 | 0.89 |
| LDL-c，mmol/l | 2.62 | 3.36 |
| TCHO，mmol/l | 4.43 | 4.86 |
| Triglyceride，mmol/l | 3.87 | 2.1 |
| Uric acid，umol/l | 438 | 278 |
| CRE，umol/l | 102 | 90 |
| hs-CRP，mg/l | 3.21 | 1.66 |
| eGFR ml/min/1.73m^2^ | 81.66 | 95.38 |
| UACR mg/g | 659.4 | 732.96 |

BMI, body mass index; FPG, fasting plasma glucose; HbA1c, hemoglobin A1c; FCP, fasting C-peptide; Fins, fasting serum insulin; TCHO, total cholesterol, LDL-c, low-density lipoprotein cholesterol; HDL-c, high-density lipoprotein cholesterol; CRE, serum creatinine; hs-CRP, high sensitivity C-reaction protein, UACR, urinary albumin/creatinine ratio.

1. One year after recruited, treatment by diet control and metformin, glimepiride, losartan.
2. Treatment by metformin, pioglitazone and losartan.

ESM Table 6 was uploaded in .xlsx format.

ESM Table 7. Classifying the rare variants of PPARG reported in previous studies according to the standards and guidelines recommended by the American College of Medical Genetics (ACMG)^30^

| Base change | Amino acid change | Functional domain | Evidence of pathogenicity | ACMG Classification | ExAc_Freq |
| --- | --- | --- | --- | --- | --- |
| GCG>GAG | p.A261E | HD | PS3^20^PM2PP2PP3PP4^20^ | Likely pathogenic | NA |
| GCT>GTT | p.A417V | LBD | PS3^22^PM1PM2PP2PP3PP4^22^ | Likely pathogenic | NA |
| TGT>CGT | p.C142R | DBD | PS3^2^PM1PM2PM6^2^PP2PP3PP4^2^ | Likely pathogenic | NA |
| TGC>TAC | p.C159Y | DBD | PS3^2^PM1PM2PP1^2^PP2PP3 | Likely pathogenic | NA |
| TGT>AGT | p.C190S | DBD | PS3^12^PM1PM2PM5^2^PP1^12^PP2PP3PP4^12^ | Likely pathogenic | NA |
| TGT>GGT | p.C190W | DBD | PS3^2^PM1PM2PP1^2^PP2PP3 | Likely pathogenic | NA |
| GAC>AAC | p.D230N | HD | PM2PP2 | Uncertain significance | NA |
| AAC>AA | p.D340K fsTer4 | LBD | PVS1PS3^2^PM1PM2PP2PP3 | Pathogenic | NA |
| GAC>AAC | p.D424N | LBD | PS3^11^PM1PM2PP2PP3PP4^11^ | Likely pathogenic | NA |
| GAT>AAT | p.D92N | AF1 | PP2PP3 | Uncertain significance | 8.25E-06 |
| GAATGT>GT | p.E138VfsTer? | AF1 | PVS1PM2PM4PP2PP3PP4^10^ | Pathogenic | NA |
| GAA>GAC | p.E157D | DBD | PS3^6^PM1PM2PP1^6^PP2PP3PP4^6^ | Likely pathogenic | NA |
| GAG>AAG | p.E217K | HD | PS3^21*^PP2PP3PP4^*^ | Likely pathogenic | 8.25E-06 |
| GAG>CAG | p.E352Q | LBD | PM1PM2PP2PP3PP4^7^ | Likely pathogenic | NA |
| GAG>AG | p.E499R fsTer12 | LBD | PVS1PM1PM2PM4PP2PP3PP4^24^ | Pathogenic | NA |
| GAA>CAA | p.E54Q | AF1 | PM2PP2 | Uncertain significance | NA |
| TTC>GTC | p.F162V | DBD | PS3^21^PM1PP2PP3 | Likely pathogenic | 8.28E-06 |
| TTT>TCT | p.F388L | LBD | PS3^9^PM1PM2PP1^9^PP2PP3PP4^9^ | Likely pathogenic | NA |
| GGT>GAT | p.G161D | DBD | PS3^21^PM1PM2PP2PP3 | Likely pathogenic | NA |
| GGT>GTT | p.G161V | DBD | PM1PM2PM5^21^PP2PP3PP4^17^ | Likely pathogenic | NA |
| CAC>CTC | p.H477L | LBD | PM1PM2PP1PP2PP3PP4^16^ | Likely pathogenic | NA |
| AAA>ACA | p.K347T | LBD | PM1PM2PP1^15^PP2PP3PP4^15^ | Likely pathogenic | NA |
| AAA>T | p.L185fsTer1 | DBD | PVS1PS3^25^PM1PM2PM4PP2PP3 | Pathogenic | NA |
| TTG>TAG | p.L339Ter | LBD | PVS1PS3^3^PM1PM2PM4PP2PP4^3^ | Pathogenic | NA |
| CTG>CCG | p.L451P | LBD | PS3^19^PM1PM2PP2PP4^19^ | Likely pathogenic | NA |
| ATG>ATC | p.M203I | DBD | PS3^22^PM1PM2PP2PP3PP4^22^ | Likely pathogenic | NA |
| ATG>TTG | p.M31L | AF1 | PM2PP2PP3PP4^22^ | Uncertain significance | NA |
| ATG>ATC | p.M376I | LBD | PS3^21^PM1PM2PP2 | Likely pathogenic | NA |
| CCA>CAA | p.P113Q | AF1 | PS3^26^PM2PP2PP3 | Likely pathogenic | NA |
| CCT>TCT | p.P387S | LBD | PS3^22^PM1PM2PP2PP3PP4^22^ | Likely pathogenic | NA |
| CCT>CTA | p.P426L | LBD | PS3^21^PM1PP2PP3 | Likely pathogenic | 8.24E-06 |
| CCG>CTG | p.P495L | LBD | PS3^4,5^PM1PM2PP1^4,5^PP2PP3PP4^4,5^ | Likely pathogenic | NA |
| CAG>TAG | p.Q121Ter | AF1 | PVS1PS3^21^PM2PM4PP2 | Pathogenic | NA |
| CGT>CAT | p.R140H | DBD | PS3^21^PM1PP2PP3 | Likely pathogenic | 8.34E-06 |
| CGG>TGG | p.R164W | DBD | PM1PM2PP2PP3 | Likely pathogenic | NA |
| AGA>ACA | p.R165T | DBD | PS3^3,21^PM1PP2PP3PP4^3^ | Likely pathogenic | 8.27E-06 |
| CGG>CAG | p.R194Q | DBD | PS3^22^PM1PM2PM4^13^PP2PP3PP4^22^ | Likely pathogenic | NA |
| CGG>TGG | p.R194W | DBD | PS3^13^PM1PM2PP2PP3PP4^13^ | Likely pathogenic | NA |
| CGG>TGG | p.R212W | HD | PS3^22^PM2PP2PP3PP4^22^ | Likely pathogenic | NA |
| CGC>CCC | p.R308P | LBD | PS3^20^PM1PM2PP2PP3PP4^20^ | Likely pathogenic | NA |
| CGA>CAA | p.R385Q | LBD | PM1PM5^2^PP2PP3PP4^22^ | Likely pathogenic | 8.29E-06 |
| CGA>TGA | p.R385Ter | LBD | PVS1PS3^2^PM1PM2PM4PP2PP4^2,28^ | Pathogenic | NA |
| CGC>TGC | p.R425C | LBD | PS3^21^PM1PM2PP2PP3PP4^1,29^ | Likely pathogenic | NA |
| TCA>TGA | p.S249Ter | HD | PVS1PS3^21^PM4PP2 | Pathogenic | 8.26E-06 |
| ACA>AGA | p.T356R | LBD | PS3^22^PM1PM2PP2PP3PP4^22^ | Likely pathogenic | NA |
| ACA>AAA | p.T468K | LBD | PS3^22^PM1PM2PP2PP3PP4^22^ | Likely pathogenic | NA |
| GTG>ATG | p.V318M | LBD | PS3^4-5,21^PM1PP2PP3PP4^4,5^ | Likely pathogenic | 8.24E-06 |
| TAT>TGT | p.Y151C | DBD | PS3^14^PM1PM2PP2PP3PP4^14,23^ | Likely pathogenic | NA |
| TAT>TGT | p.Y278C | HD | PS3^21^PM2PP2PP3 | Likely pathogenic | NA |
| TAC>TAG | p.Y355Ter | LBD | PVS1PS3^8^PM1PM2PM4PP2PP4^8^ | Pathogenic | NA |

The mutational hot spot and critical functional domains of PPARG are DNA-binding domain (DBD, protein position 140-205) and ligand-binding domain (LBD, protein position 281-505), variants at these regions had PM1 evidence. AF1, activation function 1 domain; HD, hinge domain.

NA indicates this mutation does not have a minor allele frequency (MAF) in the ExAc database.

**Supplementary figure**

**
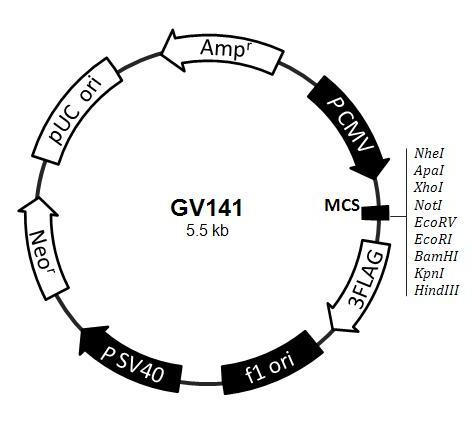
**

**ESM Figure 1. The profile of vector.** CMV-MCS-3FLAG-SV40-Neomycin. Cloning strategy: XhoI/KpnI.


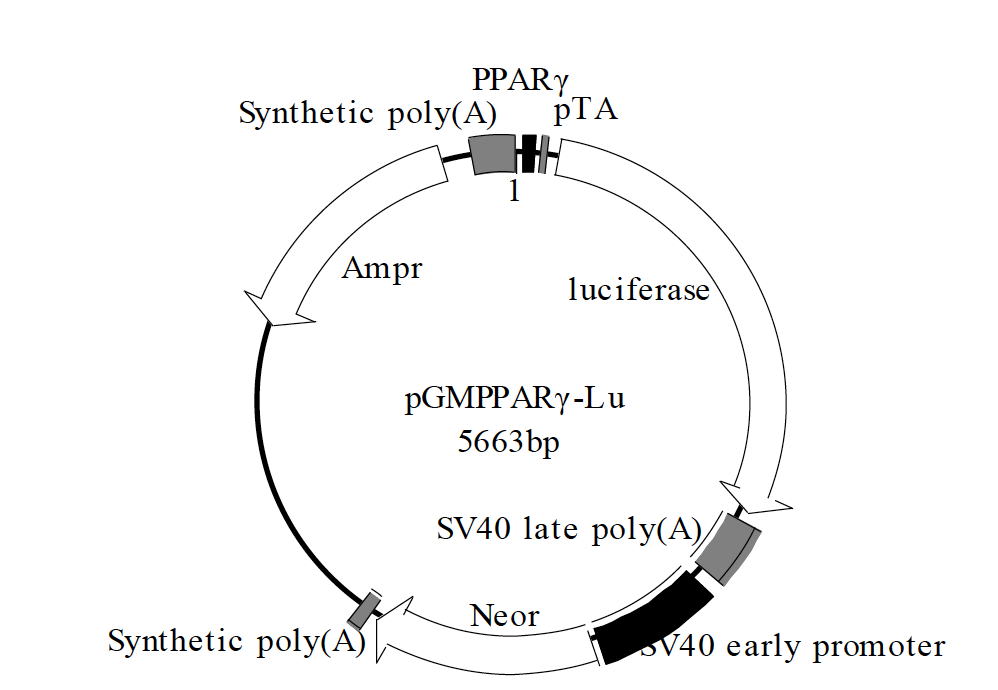


**ESM Figure 2. The profile of REPO^TM^PPARγ reporter.** PPARγ response element (PPARγ): 32-71. Minimal TA promoter (pTA): 100-122. Luciferase reporter gene: 154-1816. SV40 late poly(A) signal: 1851-2072. SV40 early promoter: 2120-2538. Synthetic neomycin phosphotransferase(Neor) coding region: 2563-3357. Synthetic poly(A) signal: 3382-3430. Synthetic Beta-lactamase(Ampr) coding region: 4545-5405. Synthetic poly(A) signal/transcriptional pause site: 5510-5663.


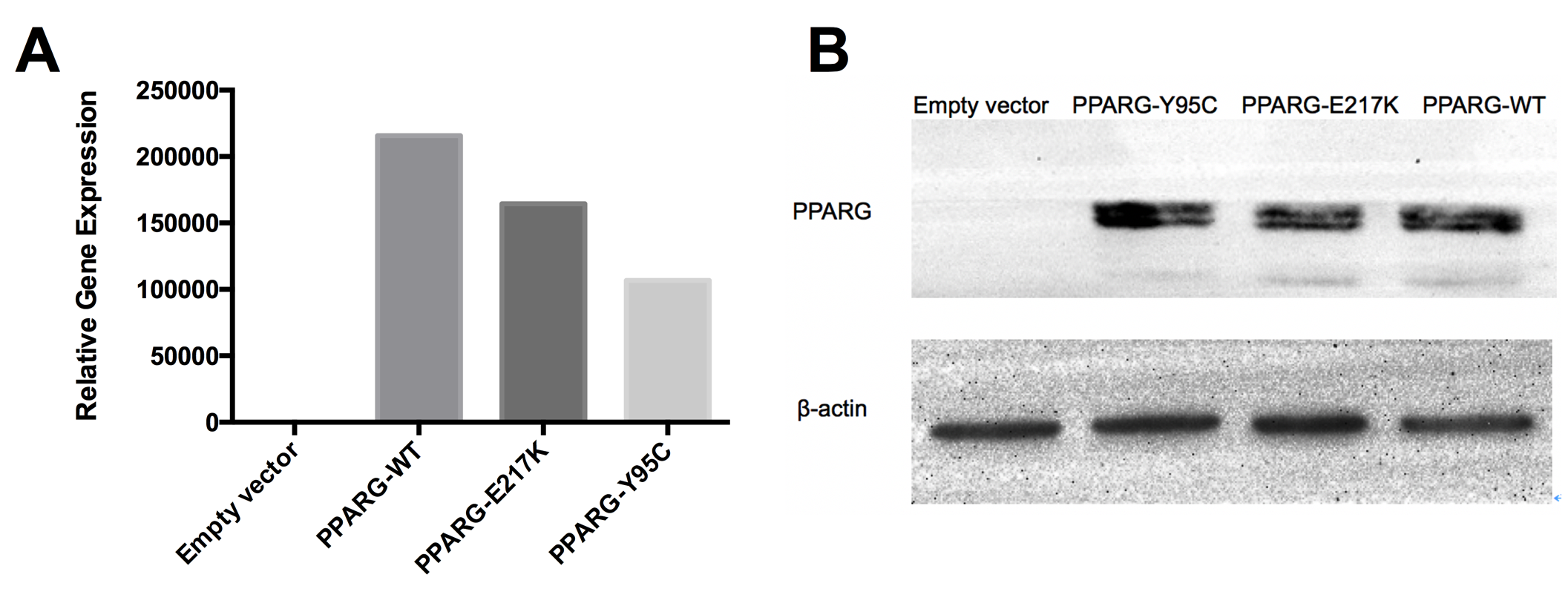


**ESM Figure 3. Gene expression of transfected 3T3-L1 preadipocytes.** Panel A, mRNA expression of human PPARG2 analyzed by quantitative PCR. Panel B, protein expression of PPARG2 analyzed by western blot.


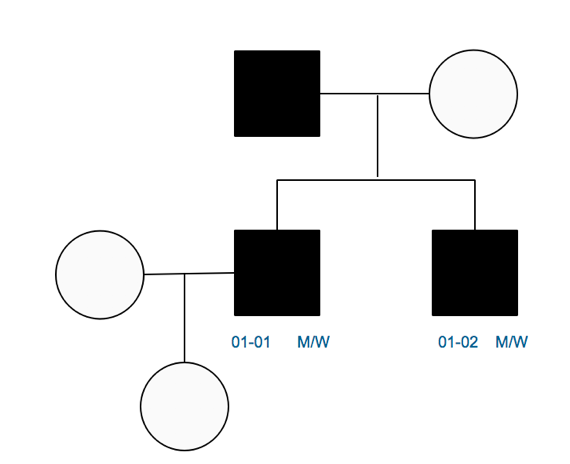


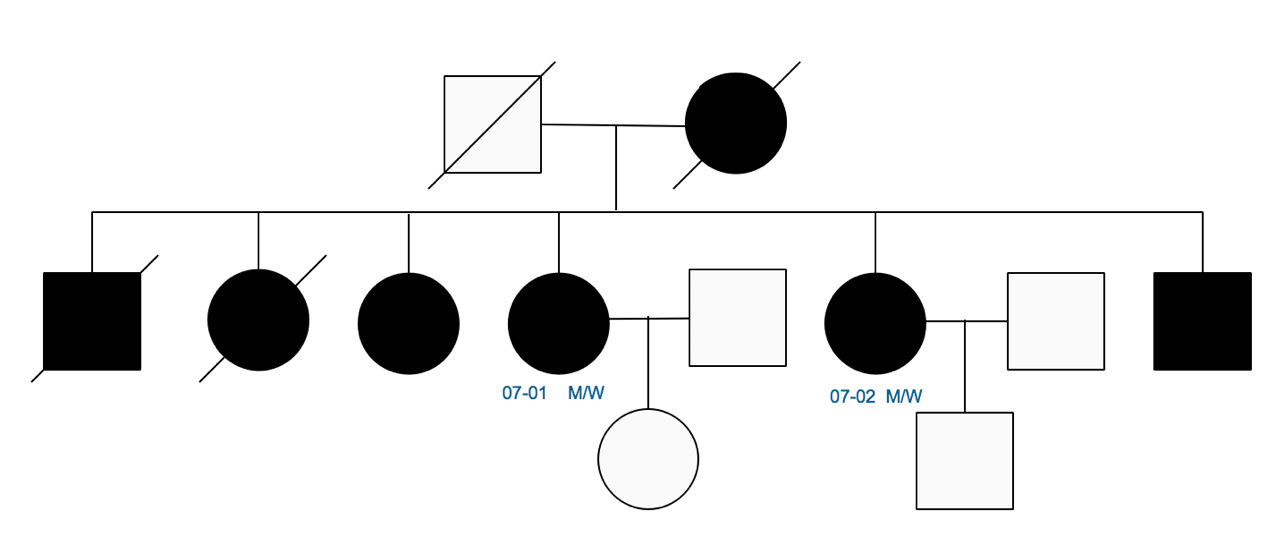


**ESM Figure 4. Pedigree of the No.01 and No.07 family.** The family members with diabetes are displayed by dark symbols. Family members carrying the heterozygote mutations are marked by M/W (M: mutation, W: wildtype).

| A | 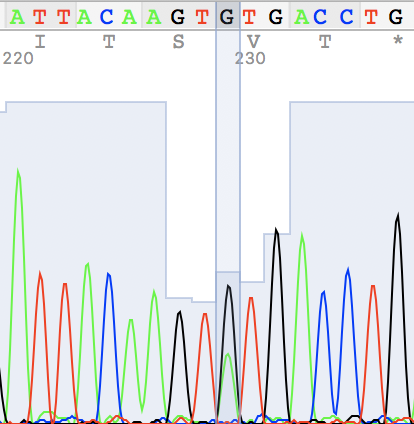 |  | 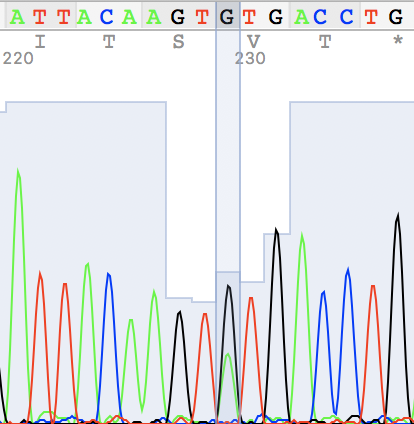 |  | 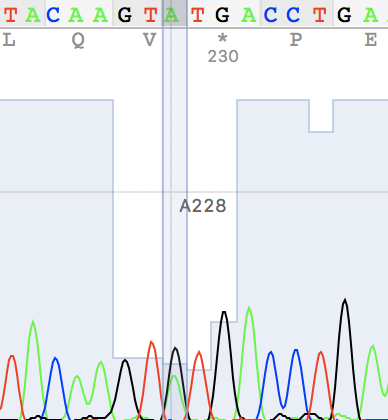 |
| --- | --- | --- | --- | --- | --- |
| B | 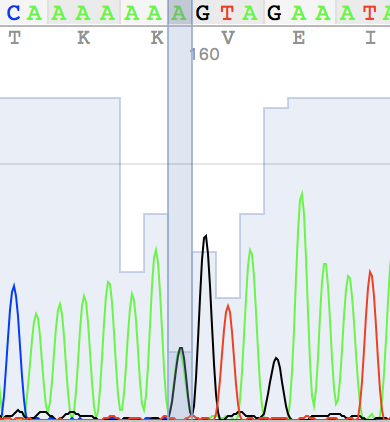 | C | 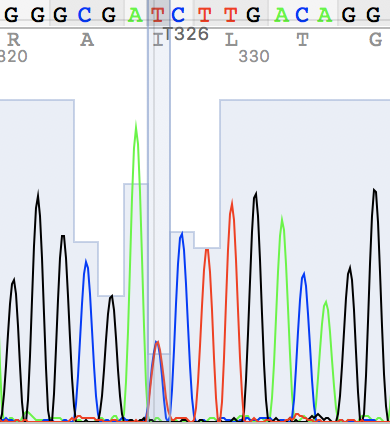 | D | 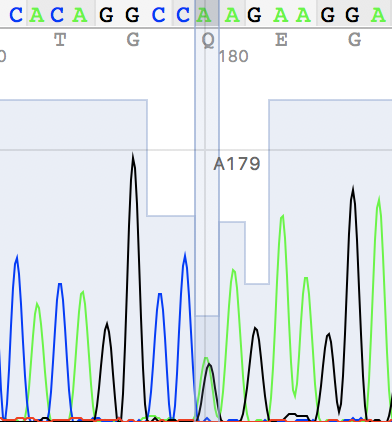 |

**ESM Figure 5. DNA sequence analysis for six patients with PPARG rare variants.** A. Heterozygous c.284A>G variants result in tyrosine-to-cystine substitutions at position 95 of the PPARG2 protein (Y95C) in three patients. B. One patient with a heterozygous c.556A>G variant leading to substitution of serine by glycine at residue 186(S186G). C. One patient with a heterozygous c.791T>C variant resulting in a isoleucine-to-threonine at position 264(I264T). D. Heterozygous c.649G>A variant leads to a substitution of glutamic acid by lysine at position 217(E217K).

**Supplementary references**

1．Agarwal AK, Garg A. A novel heterozygous mutation in peroxisome proliferator-activated receptor-gamma gene in a patient with familial partial lipodystrophy. J Clin Endocrinol Metab 2002; 87: 408–11.

2．Agostini M, Schoenmakers E, Mitchell C, et al. Non-DNA binding, dominant-negative, human PPARgamma mutations cause lipodystrophic insulin resistance. Cell Metab 2006; 4: 303–11.

3.Auclair M, Vigouroux C, Boccara F, et al. Peroxisome proliferator-activated receptor-γ mutations responsible for lipodystrophy with severe hypertension activate the cellular renin-angiotensin system. Arterioscler Thromb Vasc Biol 2013; 33: 829–38.

4.Barroso I, Gurnell M, Crowley VE, et al. Dominant negative mutations in human PPARgamma associated with severe insulin resistance, diabetes mellitus and hypertension. Nature 1999; 402: 880–3.

5.Savage DB, Tan GD, Acerini CL, et al. Human metabolic syndrome resulting from dominant-negative mutations in the nuclear receptor peroxisome proliferator-activated receptor-gamma. Diabetes 2003; 52: 910–7.

6.Campeau PM, Astapova O, Martins R, et al. Clinical and molecular characterization of a severe form of partial lipodystrophy expanding the phenotype of PPARγ deficiency. J Lipid Res 2012; 53: 1968–78.

7.Castell AL, Hiéronimus S, Lascols O, Fournier T, Fénichel P. Vascular placental abnormalities and newborn death in a pregnant diabetic woman with familial partial lipodystrophy type 3: a possible role for peroxisome proliferator-activated receptor γ. Diabetes Metab 2012; 38: 367–9.

8.Francis GA, Li G, Casey R, et al. Peroxisomal proliferator activated receptor-gamma deficiency in a Canadian kindred with familial partial lipodystrophy type 3 (FPLD3). BMC Med Genet 2006; 7: 3.

9.Hegele RA, Cao H, Frankowski C, Mathews ST, Leff T. PPARG F388L, a transactivation-deficient mutant, in familial partial lipodystrophy. Diabetes 2002; 51: 3586–90.

10.Hegele RA, Ur E, Ransom TP, Cao H. A frameshift mutation in peroxisome-proliferator-activated receptor-gamma in familial partial lipodystrophy subtype 3 (FPLD3; MIM 604367). Clin Genet 2006; 70: 360–2.

11.Lüdtke A, Buettner J, Schmidt HH-J, Worman HJ. New PPARG mutation leads to lipodystrophy and loss of protein function that is partially restored by a synthetic ligand. J Med Genet 2007; 44: e88.

12.Lüdtke A, Buettner J, Wu W, et al. Peroxisome proliferator-activated receptor-gamma C190S mutation causes partial lipodystrophy. J Clin Endocrinol Metab 2007; 92: 2248–55.

13.Monajemi H, Zhang L, Li G, et al. Familial partial lipodystrophy phenotype resulting from a single-base mutation in deoxyribonucleic acid-binding domain of peroxisome proliferator-activated receptor-gamma. J Clin Endocrinol Metab 2007; 92: 1606–12.

14.Visser ME, Kropman E, Kranendonk ME, et al. Characterisation of non-obese diabetic patients with marked insulin resistance identifies a novel familial partial lipodystrophy-associated PPARγ mutation (Y151C). Diabetologia 2011; 54: 1639–44.

15.Miehle K, Porrmann J, Mitter D, et al. Novel peroxisome proliferator-activated receptor gamma mutation in a family with familial partial lipodystrophy type 3. Clin Endocrinol (Oxf) 2016; 84: 141–8.

16.Demir T, Onay H, Savage DB, et al. Familial partial lipodystrophy linked to a novel peroxisome proliferator activator receptor -γ (PPARG) mutation, H449L: a comparison of people with this mutation and those with classic codon 482 Lamin A/C (LMNA) mutations. Diabet Med 2016; 33: 1445–50.

17.Lau E, Carvalho D, Oliveira J, Fernandes S, Freitas P. Familial partial lipodystrophy type 3: a new mutation on the PPARG gene. Hormones (Athens) 2015; 14: 317–20.

18.Dyment DA, Gibson WT, Huang L, Bassyouni H, Hegele RA, Innes AM. Biallelic mutations at PPARG cause a congenital, generalized lipodystrophy similar to the Berardinelli-Seip syndrome. Eur J Med Genet 2014; 57: 524–6.

19.Broekema MF, Massink MPG, Donato C, et al. Natural helix 9 mutants of PPARγ differently affect its transcriptional activity. Mol Metab 2019; 20: 115–27.

20.Agostini M, Schoenmakers E, Beig J, et al. A Pharmacogenetic Approach to the Treatment of Patients With PPARG Mutations. Diabetes 2018; 67: 1086–92.

21.Majithia AR, Flannick J, Shahinian P, et al. Rare variants in PPARG with decreased activity in adipocyte differentiation are associated with increased risk of type 2 diabetes. Proc Natl Acad Sci USA 2014; 111: 13127–32.

22.Majithia AR, Tsuda B, Agostini M, et al. Prospective functional classification of all possible missense variants in PPARG. Nat Genet 2016; 48: 1570–5.

23.Akinci B, Onay H, Demir T, et al. Clinical presentations, metabolic abnormalities and end-organ complications in patients with familial partial lipodystrophy. Metab Clin Exp 2017; 72: 109–19.

24.Johnston JJ, Lewis KL, Ng D, et al. Individualized iterative phenotyping for genome-wide analysis of loss-of-function mutations. Am J Hum Genet 2015; 96: 913–25.

25.Savage DB, Agostini M, Barroso I, et al. Digenic inheritance of severe insulin resistance in a human pedigree. Nat Genet 2002; 31: 379–84.

26.Ristow M, Müller-Wieland D, Pfeiffer A, Krone W, Kahn CR. Obesity associated with a mutation in a genetic regulator of adipocyte differentiation. N Engl J Med 1998; 339: 953–9.

27.Blüher M, Paschke R. Analysis of the relationship between PPAR-gamma 2 gene variants and severe insulin resistance in obese patients with impaired glucose tolerance. Exp Clin Endocrinol Diabetes 2003; 111: 85–90.

28. Johansson S, Irgens H, Chudasama KK, et al. Exome sequencing and genetic testing for MODY. PLoS ONE 2012; 7: e38050.

29. Guettier J-M, Park JY, Cochran EK, et al. Leptin therapy for partial lipodystrophy linked to a PPAR-γ mutation. Clinical Endocrinology 2008; 68: 547–54.

30. Richards S, Aziz N, Bale S, et al. Standards and guidelines for the interpretation of sequence variants: a joint consensus recommendation of the American College of Medical Genetics and Genomics and the Association for Molecular Pathology. Genet Med 2015; 17: 405–24.
